# Evolution of HD-ZIP transcription factors and their function in cabbage leafy head formation

**DOI:** 10.3389/fpls.2025.1583110

**Published:** 2025-04-03

**Authors:** Ju Zhang, Can Chen, Qihang Yang, Jie Xu, Zizhuo Han, Wei Ma, Xiaomeng Zhang, Kedong Xu, Jianjun Zhao, Xueping Chen

**Affiliations:** ^1^ State Key Laboratory of North China Crop Improvement and Regulation, Key Laboratory of Vegetable Germplasm Innovation and Utilization of Hebei, Collaborative Innovation Center of Vegetable Industry in Hebei, College of Horticulture, Hebei Agricultural University, Baoding, Hebei, China; ^2^ Key Laboratory of Plant Genetics and Molecular Breeding, Henan Key Laboratory of Crop Molecular Breeding and Bioreactor, Henan International Joint Laboratory of Translational Biology, Zhoukou Normal University, Zhoukou, Henan, China; ^3^ College of Agronomy, Henan Agricultural University, Zhengzhou, Henan, China

**Keywords:** Chinese cabbage, HD-ZIP family, phylogenetic analysis, head formation, RNA-Seq

## Abstract

**Introduction:**

The HD-ZIP protein, a unique class of transcription factors in plants, plays a crucial role in plant growth and development. Although some HD-ZIP transcription factors have been associated with leafy head formation in Chinese cabbage, their regulatory mechanisms remain poorly understood.

**Methods:**

This study identified the HD-ZIP family using HMM and TBtools, constructed a phylogenetic tree with OrthoFinder, and analyzed gene family expansion and contraction using CAFE. Conserved features were analyzed with MAFFT, MEME, and TBtools; regulatory networks were predicted using ATRM and PlantTFDB; and gene expression was validated by qRT-PCR.

**Results and discussion:**

In this study, *HD-ZIP* gene sequences from 87 species were analyzed to explore the evolutionary history of this gene family. Despite significant variation in gene family expansion and contraction across species, our findings indicated that HD-ZIP family proteins were conserved in both lower (Charophyta) and higher plants, where they were potentially involved in root, stem, and leaf differentiation. In our analysis of 22 Brassica species, HD-ZIP III protein sequences and domains were conserved. However, within the pan-genome A of 18 *Brassica rapa* species, differences were observed in auxin-related cis-elements within the HD-ZIP III promoter regions between heading and non-heading cabbage varieties. RNA-seq analysis of wild-type A03 (heading) and mutant *fg-1* (non-heading) revealed that 131 genes formed a protein interaction network or clustered in the same branch as *HD-ZIP* family genes. Through GO enrichment and qRT-PCR, several key candidate genes of *Brassica rapa* ssp. *pekinensis* A03 associated with leafy head formation in cabbage were identified. These findings established a foundation for understanding the molecular mechanisms by which the *HD-ZIP* gene family regulated head growth in Chinese cabbage.

## 1 Introduction

As the head of Chinese cabbage is its main edible organ, heading is a key agronomic trait of this plant. Accordingly, the commercial value of Chinese cabbage is directly determined by head quality (Ren et al., 2020). Chinese cabbage heads do not form in plants harboring mutations in the leafy microtubule arrangement gene *BrAN* (Xin et al., 2022), the gibberellic acid (GA) biosynthesis-related genes *BrCPS1* (Gao et al., 2022) and *BrKAO2* (Huang et al., 2022), or the leafy head formation gene *BrLFM* (Zhao et al., 2023). Other genes (including some transcription factor genes) have also been identified as regulating head formation and development in Chinese cabbage. For example, the *BcpLH* gene causes changes in head size (Ren et al., 2020), and the *BrpSPL9* gene controls the heading time (Wang et al., 2014). *CYCLOIDEA AND PCF TRANSCRIPTION FACTOR* genes modulate head shape (Mao et al., 2014). The auxin-response factors ARF3 and ARF4 (Cheng et al., 2016), and Homeodomain leucine zipper III (HD-ZIP III) family belong to key regulators of leafy adaxial/abaxial characteristics (Cheng et al., 2016).

HD-ZIP proteins are transcription factors that are unique to plants (Elhiti and Stasolla, 2009). All HD-ZIP transcription factors consist of two conserved domains, the homologous box domain (HD) and the homeobox associated basic leucine zipper (bZIP) element, which is tightly connected to the carboxy terminus of HD (Li et al., 2022). The HD domain is involved in DNA binding through a helix-turn-helix (HTH) structure, whereas bZIP is related to protein dimerization (Elhiti and Stasolla, 2009). The HD-ZIP transcription factor family consists of four subfamilies, namely, HD-ZIP I, HD-ZIP II, HD-ZIP III, and HD-ZIP IV (Elhiti and Stasolla, 2009; Sharif et al., 2021). These four subfamilies have distinct biological functions in plants. Many HD-Zip I members have been demonstrated to play important roles in plant growth regulation (Li et al., 2023, 2019a) [e.g., promoting tomato fruit ripening (Li et al., 2023)] and regulate responses to various abiotic stressors (Ebrahimian-Motlagh et al., 2017; Gong et al., 2019; Wu et al., 2022; Zhang et al., 2012; Żyła and Babula-Skowrońska, 2023) [e.g., responding to abscisic acid (Zhang et al., 2012), drought (Ebrahimian-Motlagh et al., 2017; Zhang et al., 2012), cold (Zhang et al., 2012), and oxidative stress (Żyła and Babula-Skowrońska, 2023)]. HD-ZIP II members play roles in plant growth by responding to hormones, such as auxin (He et al., 2020; Possenti et al., 2024; Turchi et al., 2015), ethylene (Gu et al., 2019), and gibberellin (Chen et al., 2019). They also contribute to symmetry (Carabelli et al., 2021), leaf development (Bou-Torrent et al., 2012), and drought resistance (Sasaki et al., 2019; Zhu et al., 2025). The HD-ZIP III subfamily proteins have critical functions in the establishment of adaxial/abaxial polarity in plant leaves, and miR165/166 directly regulates their expression by degrading their mRNAs (Merelo et al., 2016). In rice and cucumber, a base mutation in the complementary miR165/166 binding site of the *PHABULOSA* (*PHB*) gene results in a functional acquisition curled leaf mutant phenotype. The phenotype of rice overexpressing *HOMEOBOX GENE 3* (*OSHB3*, a homologous gene of *Arabidopsis thaliana PHB* and *PHAVOLUTA (PHV)*) remains unchanged from the wild type. However, when 5 base synonymous changes occur at the *OSHB3* and miR165/166 binding sites, rice leaves curl (Itoh et al., 2008). In cucumber, ethylmethylsulfone generates two functionally acquired mutants: *cul-1* and *cul-2*, both of which demonstrated inward leaf curving, and the mutation sites of *cul-1* and *cul-2* have been identified at the miRNA165/166 complementary site of *CsPHB*. In many organs of the *cul-1* and *cul-2* mutants, *CsPHB* expression is higher than that in the wild type (Rong et al., 2019). The *LEAFY HEADS* (*BcpLH*) gene directly binds to and participates in the cleavage of pri-miRNA during head formation in Chinese cabbage, forming the *BcpLH*–miR165/166–*HD-ZIP III* module to regulate the polarity development of Chinese cabbage leaves (Ren et al., 2020; Liu et al., 2011). The functions of HD-ZIP IV members are diverse. They can drive the differentiation of epidermal cell types (Schrick et al., 2023), regulate male flowering time and fertility in cucumber (Cai et al., 2020), cooperatively promote the development of interconnecting trichomes at the tomato anther margin (Wu et al., 2024), have functions in plant growth and defense against adverse environmental influences (Chew et al., 2013), inhibit anthocyanin biosynthesis in strawberry fruit (Sun et al., 2025), and regulate the lignin content (Sun et al., 2020). In summary, HD-ZIP proteins regulate the growth, development, and abiotic stress responses in plants by modulating downstream target genes and hormone regulatory networks (Li et al., 2022).

The assembly quality of plant genome sequencing has significantly improved with advancements in genome resequencing technology, and this enables more in-depth study of gene evolution. Studying the evolution and abundance of the *HD-ZIP* family from algae to eukaryotes can deepen our understanding of the plant *HD-ZIP* gene family. Although it is known that *HD-ZIP* family members are involved in heading development in Chinese cabbage, the detailed molecular mechanisms are unclear. Therefore, in this study we conducted evolutionary and bioinformatics analyses on the *HD-ZIP* family in organisms ranging from algae to eukaryotes. In our previous study, we obtained transcriptome data from a flat leaf-growing non-heading mutant (*fg-1*) induced by EMS and the wild-type heading cabbage A03 (Li et al., 2019b). In this study, these transcriptome data were used to determine the differential expression of *HD-ZIP* family genes in Chinese cabbage during head development. Our results provide a foundation for dissecting the precise mechanism by which the *HD-ZIP* gene family is involved in Chinese cabbage leafy head development.

## 2 Materials and methods

### 2.1 Collection and identification of HD-ZIP proteins

HD-ZIP protein sequences were identified from genomic and pan-genomic databases, which were downloaded from Phytozome (https://phytozome-next.jgi.doe.gov/), NCBI (https://www.ncbi.nlm.nih.gov/), (https://plants.ensembl.org/index.html), BRAD (http://www.brassicadb.cn/#/), NCCWDB (http://tbir.njau.edu.cn/NhCCDbHubs/index.jsp), and CCEMD (http://www.bioinformaticslab.cn/EMSmutation/home/). The Hidden Markov Model (HMM) profiles of the HD (PF00046), bZIP (PF02183), and SMART (PF01852) domains of HD-ZIP were obtained from InterProScan (https://www.ebi.ac.uk/interpro/). TBtools-II (v2.030) (Chen et al., 2020) was used to identify HD-ZIP family members by querying three HMM profiles against the protein sequences of each plant species (E-value < 0.01). The candidate HD-ZIP proteins were further verified using SMART (https://smart.embl.de/) and InterProScan (https://www.ebi.ac.uk/interpro/). Evolutionary timescales were predicted for all plant species using Timetree of Life (http://www.timetree.org/).

### 2.2 Building an evolutionary tree with multiple species

Using OrthoFinder’s default settings, a species evolutionary tree was built. In brief, we gathered the whole genome sequences of several species and entered the protein sequences into OrthoFinder as input files. After using default BLAST or DIAMOND (for the sequence similarity search) to identify orthogonal genes between species, OrthoFinder was used to cluster the orthogonal genes using the default Markov clustering algorithm (MCL) and built an evolutionary tree for the species based on the orthogonal gene family. The evolutionary tree was constructed using the STAG method for phylogenetic inference with branch lengths determined by the average replacement rate between genes. The output was in Newick format.

### 2.3 Gene expansion and contraction in the genomes of different species

To estimate gene family expansion and contraction, CAFE was used with default settings to obtain expansion and contraction rates (lambda) of gene families based on the species evolutionary tree. Based on the phylogenetic trees, CAFE implemented a random walk model that assumed all gene families had the same rate of growth and contraction (lambda). In the calculation, CAFE first read the input gene family count file and combined it with the phylogenetic tree structure to identify changes in gene number. The maximum likelihood estimation method was employed for this analysis in each gene family on the species branch.

### 2.4 Conserved domain, conserved motif, and chromosomal location analyses of the HD-ZIP family

Using MAFFT online version 7 (https://mafft.cbrc.jp/alignment/software/), multi-sequence alignments of HD-ZIP III protein sequences in 22 Brassicaceae plants were generated. The tree was subsequently trimmed using iTOL v6 (https://itol.embl.de/). Conserved motifs were identified using MEME (https://meme-suite.org/meme/tools/meme). The NCBI Batch CD-Search function (https://www.ncbi.nlm.nih.gov/Structure/bwrpsb/bwrpsb.cgi) was used to analyze the protein sequences for conserved domains of multiple protein sequences. TBtools was used to determine the chromosomal positions of HD-ZIP III proteins. All results were visualized using TBtools or iTOL v6.

### 2.5 Interspecies synteny analysis and gene duplication

To analyze the genetic relationships of different HD-ZIPs in *Brassica rapa* ssp. *pekinensis* A03, multiple sequence alignments were performed to detect the coding sequences (CDSs) with a similarity degree of more than 70%. The Multiple Collinearity Scan Toolkit (MCScanX; https://github.com/wyp1125/MCScanX) was used to analyze the collinear regions with default parameters. The synteny analysis map of HD-ZIPs in *Brassica rapa* ssp. *pekinensis* A03 was illustrated using Python package JCVI (https://github.com/tanghaibao/jcvi). Gene duplication analysis was performed using the MCScanX program with default parameters, and the location and relationship of duplicated genes were determined using Circos software.

### 2.6 Predictive network of *BaaHD-ZIP* genes involved in cabbage heading

Upstream and downstream regulatory genes of *BaaHD-ZIPs* were predicted and analyzed in *Brassica rapa* ssp. *pekinensis* A03 using ATRM (https://atrm.gao-lab.org/index.php), PlantTFDB (https://planttfdb.gao-lab.org/index.php), PlantRegMap (https://plantregmap.gao-lab.org/index.php), and STRING (https://cn.string-db.org/). Heat map analysis and Gene Ontology (GO) enrichment analysis were performed in RStudio 4.3.2.

### 2.7 Total RNA extraction and qRT-PCR

At the early heading stage (80 days after sowing) of Chinese cabbage (the mutant *fg-1* and the wild-type A03), samples were collected from the base of the 16th leaf petiole from the exterior of the growing head. Three replicates of the tests were carried out, for each sample, and each replicate contained three technical replicates. RNA extraction, quantitative real-time PCR (qRT-PCR), and reverse transcription were carried out as previously described (Zhang et al., 2017). Data were computed using the 2^-ΔΔCT^ method. Primer sequences for detecting *BaaHB-8*.*1*, *BaaHB-8*.*2*, *BaaREV*.*1*, *BaaREV*.*2*, *BaaPHV*, *BaaREV*.*3*, *BaaHB-15*.*1*, *BaaPHB*.*1*, *BaaPHB*.*2*, *BaaHB-15*.*2*, *BaaARF5*, *BaaZPR1*, *BaaAS2*, *BaaAS1*.*1*, and *BaaAS1*.*2* are listed in **Supplementary Table S1**. *Baa-Actin* was used as an internal control.

## 3 Results

### 3.1 HD-ZIP transcription factors and plant evolution

#### 3.1.1 Analysis of *HD-ZIP* family genes during the evolution of algal and land plant species

**Figure 1:**
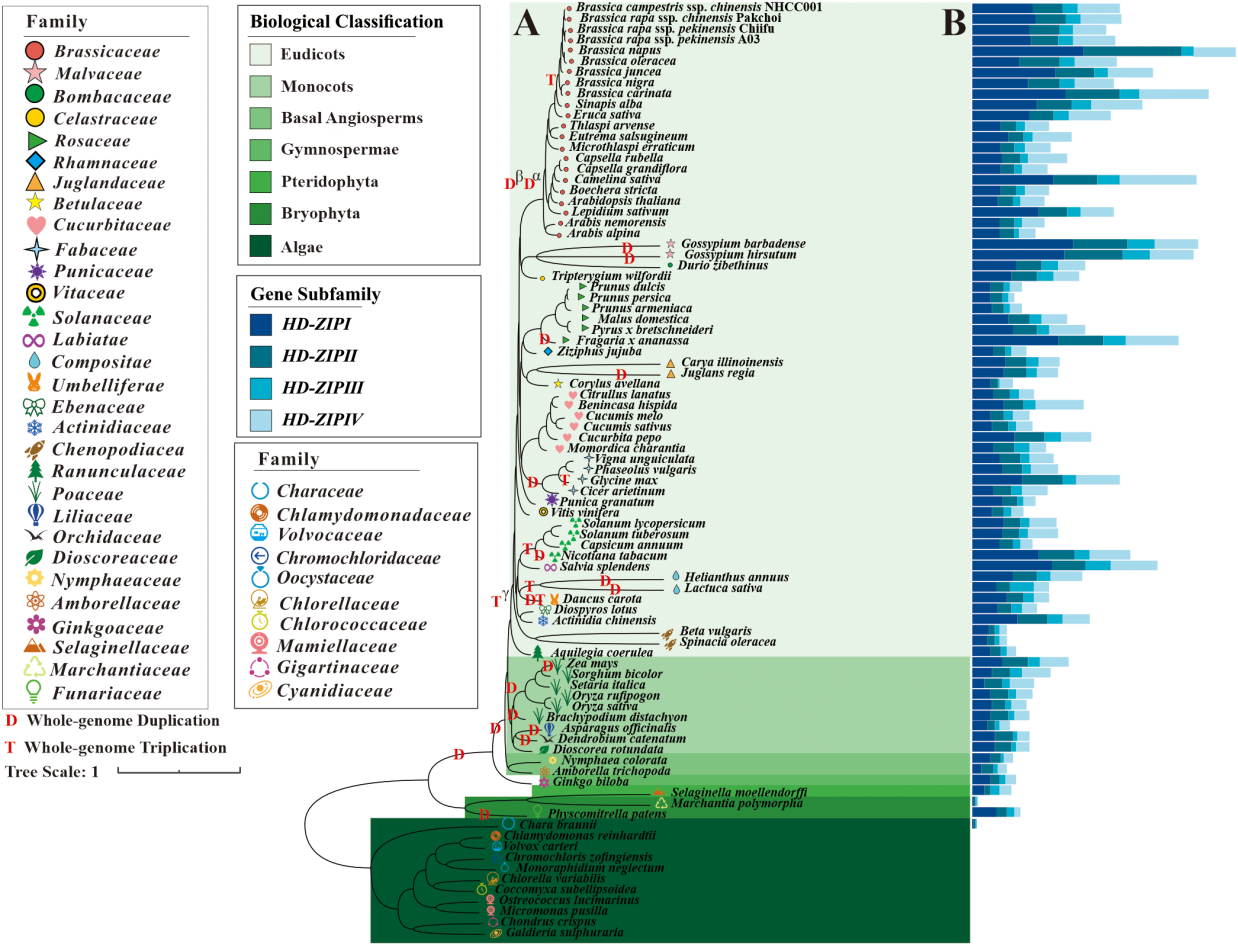
Numbers of *HD-ZIP* genes in different clade of phylogenetic tree. **(A)** Phylogenetic tree of 87 plant species; **(B)** Gene numbers of *HD-ZIP I*, *HD-ZIP II*, *HD-ZIP III*, and *HD-ZIP IV* subgene families in each species’ genomes.

*HD-ZIP* family genes were comprehensively identified in 87 species (**Supplementary Table S2**). These species represented different clades of the evolutionary tree in the plant kingdom, including algae, bryophytes, pteridophytes, gymnosperms, basal angiosperms, monocots, and eudicots. A total of 4848 *HD-ZIP* family genes were identified in 40 species (**Supplementary Table S2**). Following a screening process, no *HD-ZIP* genes were detected in the 10 algal plants of the Rhodophyta and Chlorophyta clades emerged between 1161.0 and 88.2 million years ago (MYA) (**Figure 1**; **Supplementary Figure S1**). Only three *HD-ZIP* genes—one *HD-ZIP I*, one *HD-ZIP II*, and one *HD-ZIP III* subfamily gene—were found in *Chara braunii* of the Charophyta clade emerged around 942.7 MYA (**Figure 1**; **Supplementary Figure S1**). In the evolutionary history of approximately 460.0–497.3 MYA, *HD-ZIP* family genes were identified in 77 embryogenic plants, with each species having 4 *HD-ZIP* subfamily genes (**Figure 1**; **Supplementary Figure S1**). The moss *Physcomitrella patens* genome harbored 33 *HD-ZIP* family genes, with 17 from the *HD-ZIP I* subfamily, 7 from the *HD-ZIP II* subfamily, 5 from the *HD-ZIP III* subfamily, and 4 from the *HD-ZIP IV* subfamily. *Marchantia polymorpha*, a moss, had four *HD-ZIP* family genes, and each of the four *HD-ZIP* subfamilies included only one gene. Pteridophyte *Selaginella moellendorffi* had 8 *HD-ZIP I* genes, 4 *HD-ZIP II* genes, 6 *HD-ZIP III* genes, and 8 *HD-ZIP IV* genes out of a total of 26 *HD-ZIP* genes (**Figure 1**; **Supplementary Figure S1**). Gymnosperms began to branch off from the plant evolutionary tree at 330.3 MYA. *Ginkgo biloba*, one early gymnosperm species, had 12 *HD-ZIP I* genes, 6 *HD-ZIP II* genes, 4 *HD-ZIP III* genes, and 7 *HD-ZIP IV* genes. The appearance of monocots could be traced back to about 120.3 MYA. In the 9 monocotyledonous plants in this study, including *Zea mays* (corn), *Brachypodium distachyon* (setaria), *Oryza rufipogon* (wild rice), and *Asparagus officinalis* (asparagus), there were 25–65 *HD-ZIP* family genes per species.

Dicotyledonous plants first appeared approximately 129.3 MYA. The number of *HD-ZIP* genes among the 61 dicotyledonous plants ranged from 23 to 175. There were 89–175 *HD-ZIP* family genes in the 9 *Brassica* species or variants, and *Brassica* plants had a higher number of *HD-ZIP* family genes than other genera and species (**Figure 1**; **Supplementary Figure S1**). Even in *Arabidopsis*, which shares an ancestor with *Brassica* from 25.97 MYA, there were just 48 *HD-ZIP* family genes. The reason for this could be that, in addition to the ancient genome polyploidization events of T^γ^, D^β^, and D^a^ shared by the Brassicaceae family, the *Brassica* genus also experienced a more recent whole genome triploidization event (WGT) around 10 MYA (**Figure 1**). In addition to the Brassicaceae family, many major families, including Poaceae, Fabaceae, Compositae, and Solanaceae, also underwent such polyploidization events.

#### 3.1.2 Gene family expansion and contraction among nine plant species

**Figure 2:**
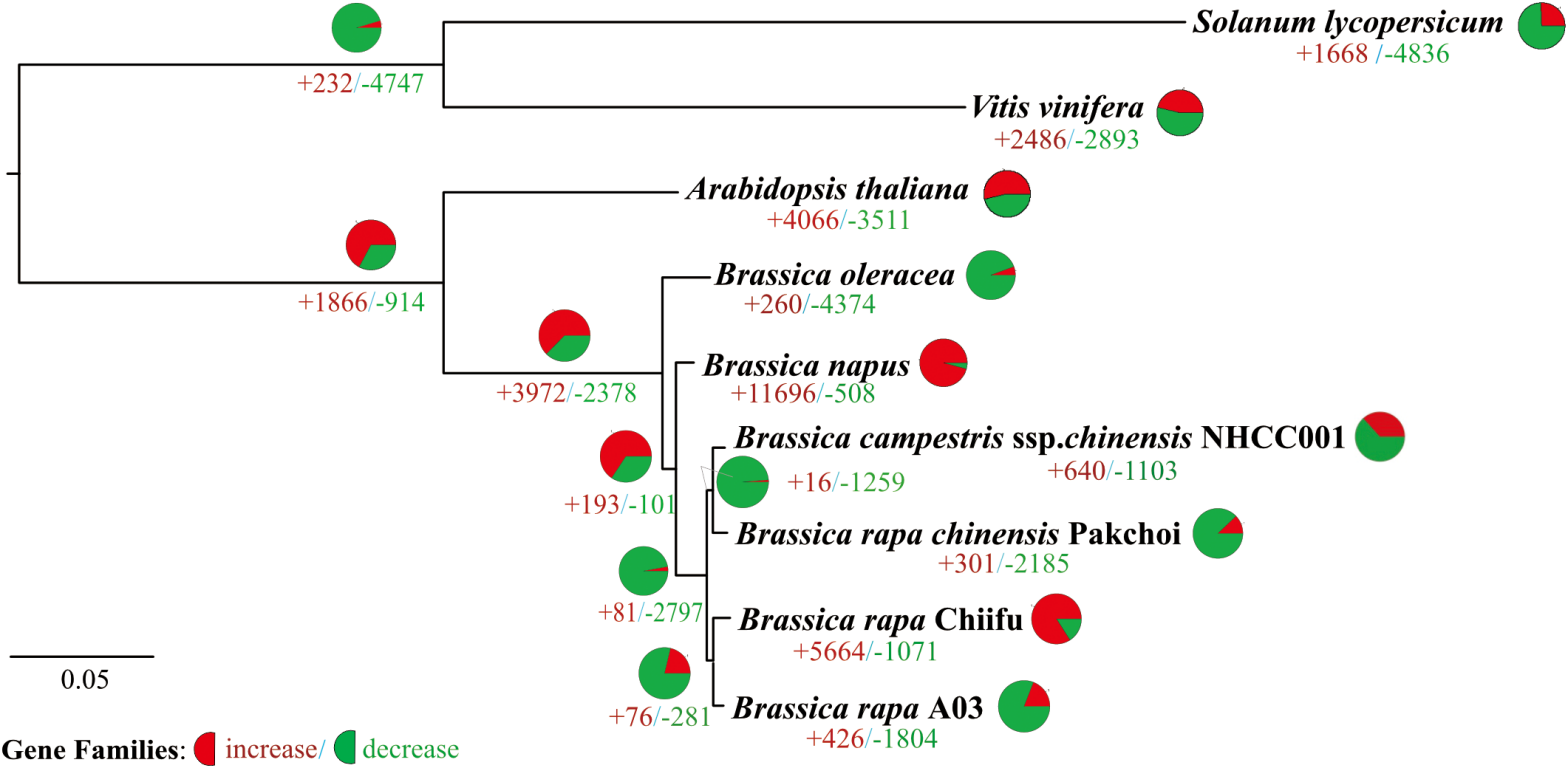
Phylogenetic relationships and number of gene families displaying expansion and contraction among nine selected plant species. Pie diagram on each branch of the tree represents the proportion of genes undergoing expansion (red) or contraction (green) events. And the ‘+ number’ (red) represent the number of expanded gene families, the ‘- number’ (green) represent the number of contracted gene families.

To analyze the expansion and contraction of gene families, the changes in the number of gene family members were calculated among the genomes of *Solanum lycopersicum*, *Vitis viniferagrape*, *Arabidopsis thaliana*, *Brassica oleracea*, *Brassica napus*, and four types of cabbage (including two heading cabbages, Chiifu and A03, and two non-heading cabbages, NHCC001 and Pakchoi). The results are visualized in **Figure 2**, which each clade of the evolutionary tree displayed gene family expansion and contraction. In the Brassicaceae clade, there were 914 gene family contractions and 1866 gene family expansions. According to the inference results, there was an absence of 2378 families and an addition of 3972 families in the *Brassica* clade (**Figure 2**). Except for heading cabbage in the Chiifu clade, the number of expanded gene families was lower than the number of contracted families in other clades of *Brassica rapa*. Gene family expansion and contraction varied significantly between the heading cabbage Chiifu and A03 clades and between the non-heading cabbage NHCC001 and pakchoi clades. Of the non-heading cabbage clades, 16 gene families were expanded and 1259 were contracted; the NHCC001 clade had 640 expanded and 1103 contracted families, while pakchoi had 301 expanded and 2158 contracted families. There were differences in the number of expanded and contracted families between the two headed cabbage clades. The A03 clade included 1814 contracted and 426 expanded gene families, compared to 5664 expanded and 1071 contracted families in the Chiifu clade (**Figure 2**).

### 3.2 Analysis of HD-ZIP proteins in the Brassicaceae genome and pan-genome A

#### 3.2.1 Identification of HD-ZIP protein family members in Brassicaceae

**Figure 3:**
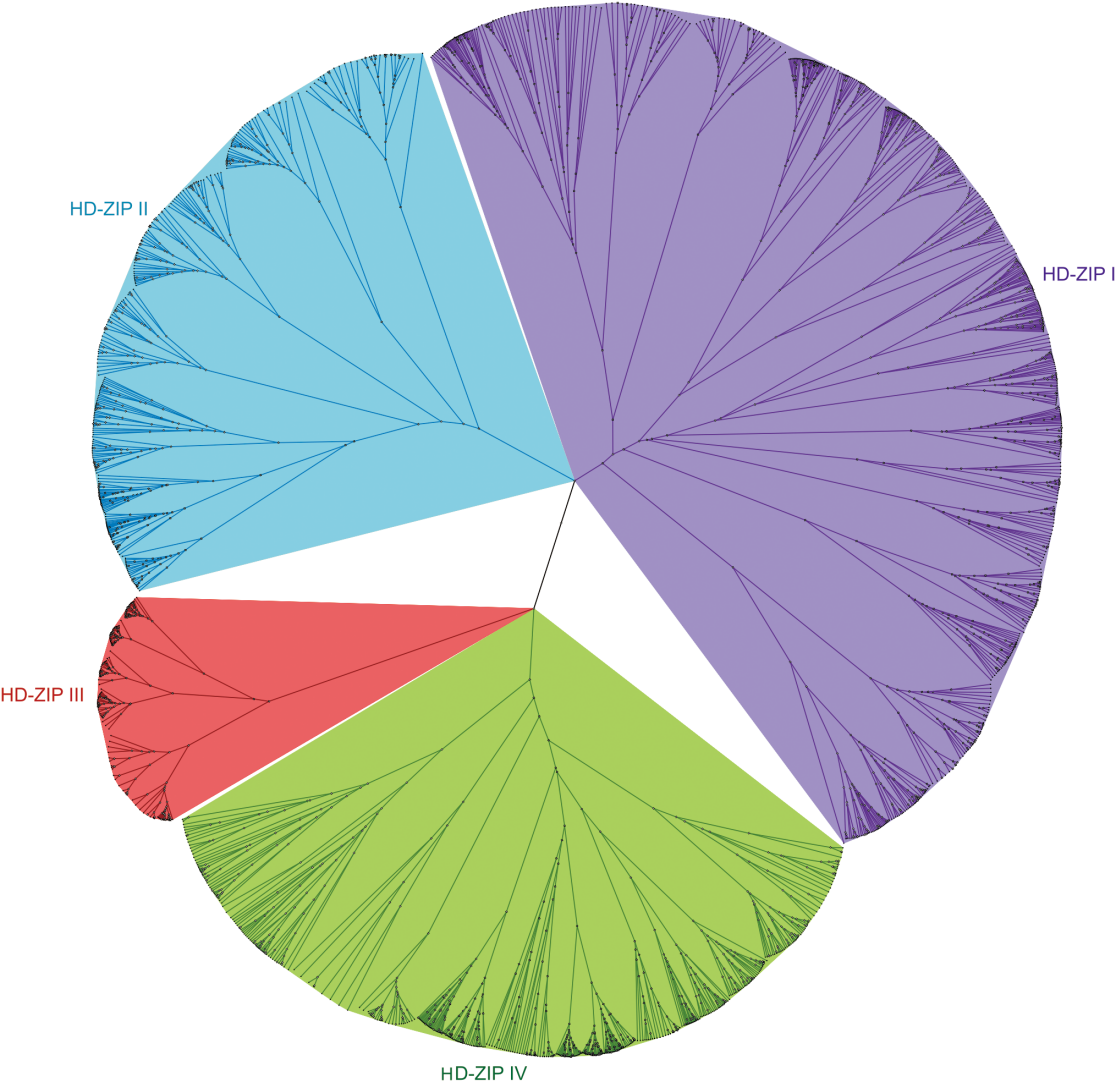
Phylogenetic tree of HD-ZIP protein family members in 22 Brassicaceae species. In the genomes of 22 selected Brassicaceae species, 1940 proteins from the HD-ZIP family were identified: HD-ZIP I—781 proteins, HD-ZIP II—380 proteins, HD-ZIP III—192 proteins, and HD-ZIP IV—587 proteins.

In the genomes of 22 selected Brassicaceae species, 1940 proteins from the HD-ZIP family were identified, and in the phylogenetic tree, they were clustered into 4 subfamilies: HD-ZIP I, HD-ZIP II, HD-ZIP III, and HD-ZIP IV (**Figure 3**). Among them, HD-ZIP I had the highest number of proteins, with 781, followed by HD-ZIP IV with 587, HD-ZIP II with 380, and HD-ZIP III with 192. HD-ZIP III was also the most conservative subfamily according to the tree scale (**Figure 3**; **Supplementary Table S3**). Of the 22 Brassicaceae species, the HD-ZIP family of *Brassica napus* has the largest number of HD-ZIP proteins (175), whereas the HD-ZIP family of *Arabis alpina* contained the fewest, with only 41 proteins. The heading cabbage varieties *Brassica rapa* ssp. *pekinensis* A03 and Chiifu contained 95 and 89 HD-ZIP family proteins, respectively. The two varieties of non-heading cabbage, *Brassica campestris* ssp. *chinensis* NHCC001 and *Brassica rapa* ssp. *chinensis* Pakchoi, had 98 and 99 HD-ZIP family proteins, respectively (**Supplementary Table S3**).

#### 3.2.2 Identification of conserved domains and motifs in Brassicaceae HD-ZIP III subfamily proteins

**Figure 4:**
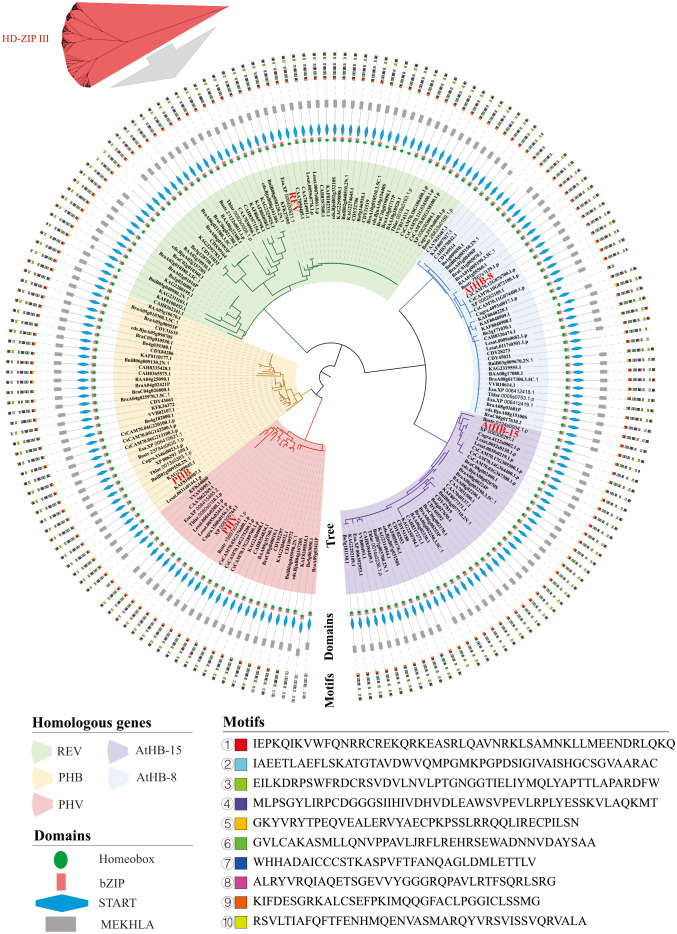
Conserved domains and motifs of HD-ZIP III proteins in 22 Brassicaceae species. In 22 Brassicaceae species, the 192 proteins were grouped in five branches and clustered with the following proteins in *Arabidopsis thaliana*: REV (57 proteins), PHB (33 proteins), PHV (26 proteins), AtHB-8 (37 proteins), and AtHB-15 (39 proteins).

The similarity and diversity of conserved domains and motifs were determined in HD-ZIP III proteins (**Figure 4**). As shown in **Figure 4**, these 192 proteins were grouped into five branches and clustered with REV (57 proteins), PHB (33 proteins), PHV (26 proteins), AtHB-8 (37 proteins), and AtHB-15 (39 proteins) in *Arabidopsis thaliana*. The protein domains of the HD-ZIP III subfamily were highly conserved in the 22 Brassicaceae species (**Figure 4**), and most proteins had four domains: homeobox, bZIP, START, and MEKHLA. However, a very small number of proteins lost the MEKHLA domain. Five such proteins were found in *Brassica napus* (CDY31032, CDY51188, CDY28273, CDY45021, and CDY69514), two in *Brassica oleracea* (Bo1g009850.1 and Bo3g128750.1), and one each in *Arabidopsis thaliana* (AtHB-15), *Microtlaspi erraticum* (CAA7062369.1), and *Sinapis alba* (KAF8048228.1). The motifs of the HD-ZIP III subfamily were likewise very conserved. **Figure 4** shows the first ten motifs. In our analysis, all proteins grouped in the same branch as AtHB-15 lacked ‘motif ⑧’. Ten motifs were present in the majority of the proteins in the four branches aggregated with REV, PHB, PHV, and AtHB-8. Similar to the situation that some proteins lacking the MEKHLA domain, some motifs were lost in BniB05g040980.2N.1 from *Brassica nigra*, XP 006283109.1 from *Capsella rubra*, CAH8362341.1 from *Eruca sativa*, and KFK36372 from *Arabis alpina*. There were also some proteins with most motifs lost. For example, only three motifs (motifs ⑤, ①, and ②) were present in *Sinapis alba* KAF8048228.1, and it was the protein with the highest degree of motif losses. In addition, the CAA7062369.1 protein in *Microtlaspi erraticum* had only 4 motifs (motifs ⑤, ①, ②, and ③), representing the second most motif-missing protein. Based on the analysis results in **Figure 4**, the C-terminus of the HD-ZIP III subfamily was highly conserved, while the N-terminus showed more variation. Some proteins were characterized by MEKHLA domain deletions and partial motif deletions at the N-terminus.

#### 3.2.3 Analysis of *cis*-acting elements in the HD-ZIP III promoters within the pan-genome

**Figure 5:**
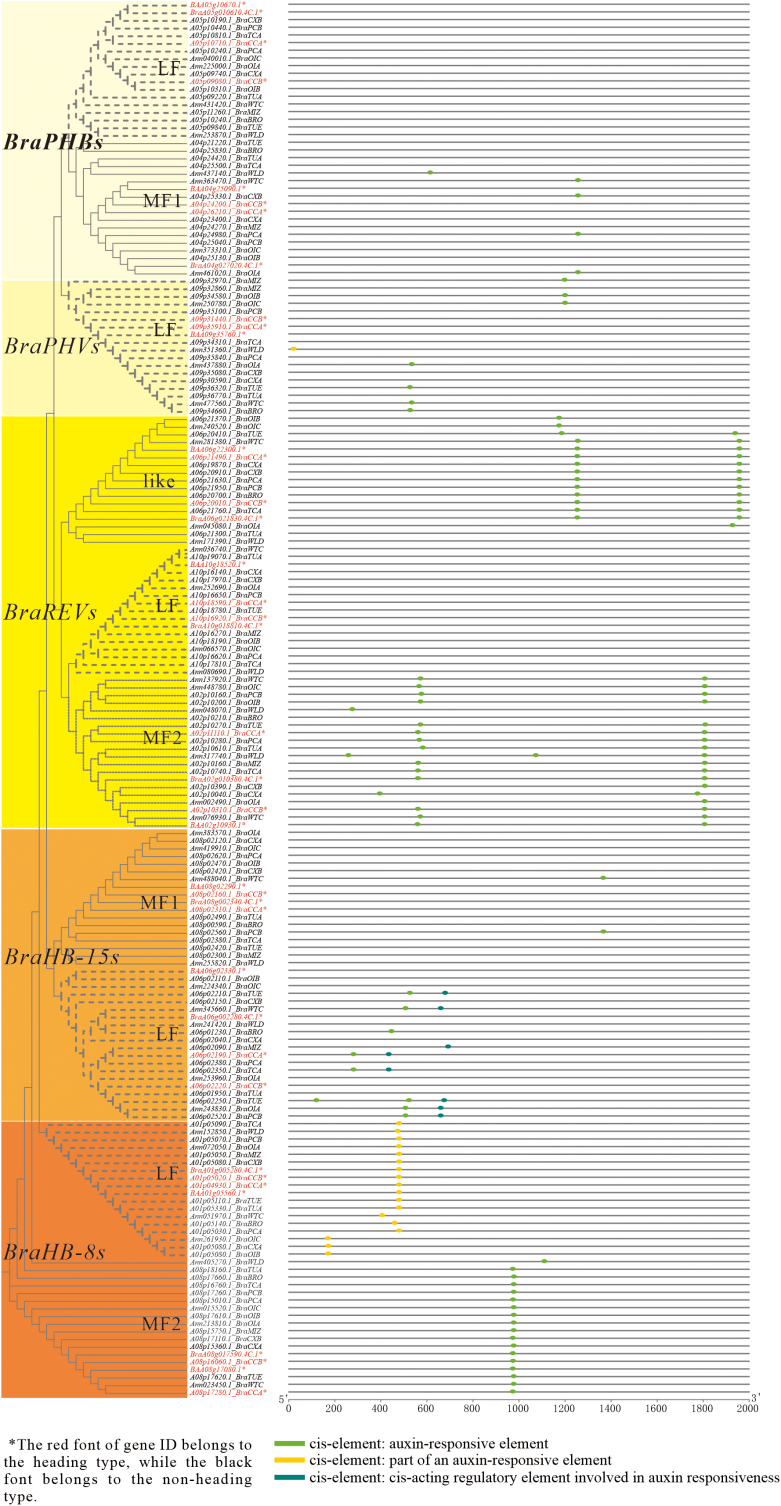
Comparative analysis of *HD-ZIP III* promoter *cis*-acting elements in the pan-genome. By BLAST tools for homology annotation in BRAD database, LF represents the least fractioned subgenome, and MFs (MF1 and MF2) represent more fractioned subgenomes.

In order to understand the important functions of the *HD-ZIP III* gene subfamily in the development of heading and non-heading cabbage, this research identified the HD-ZIP III subfamily proteins in the pan-genome of 18 *Brassica rapa* accessions (all belonging to the A genome) (Cheng et al., 2016). These proteins contain five homologous genes: *BraPHBs*, *BraPHVs*, *BraREVs*, *BraHB-8s*, and *BraHB-15s*. Furthermore, their protein sequences and conserved domains are highly conserved (not shown in the article). However, through analysis of their promoter *cis*-acting elements, it was found that the distribution of auxin-related *cis*-elements differed the most compared to those related to gibberellin, abscisic acid, salicylic acid, and methyl jasmonate (MeJA) (**Supplementary Table S4**). Three different kinds of auxin-related *cis*-elements were present in these promoter sequences: an auxin-responsive element (*cis*-element ①), part of an auxin-responsive element (*cis*-element ②), and a *cis*-acting regulatory element involved in auxin responsiveness (*cis*-element ③) (**Figure 5**). The promoters of all *BraREVs-like* and *BraREVs-MF2* genes contained one or two *cis*-elements ①. Only the promoters of the *BraHB-8s-LF* genes had *cis*-element ②, with the exception of gene *A09p35100.1-BraPCB* (*BraPHVs-LF*). The *BraHB-15s-LF* promoters contained both *cis*-element ① and *cis*-element ③, with nearly all of these genes found in non-heading cabbage. One *cis*-element ① was present in some promoters of *BraPHBs-MF1*, *BraPHVs-LF*, and *BraHB-15s-MF1*, and nearly all of these promoters belonged to non-heading cabbage. In contrast, the *BraPHBs-LF* and *BraREVs-LF* gene promoters did not contain any of the three *cis*-elements ①–③. Thus, while the HD-ZIP III subfamily protein sequences were highly conserved, the distribution of these auxin-related *cis*-elements in their promoter regions differed greatly, as did the gene promoters of heading and non-heading cabbage, according to the analysis of 18 *Brassica rapa* pan-genomes. It is speculated that HD-ZIP III is involved in the heading development process of Chinese cabbage under the influence of auxin.

### 3.3 Transcriptome-based analysis of HD-ZIP proteins in cabbage leafy head formation

#### 3.3.1 Chromosomal localization, collinearity and *cis*-acting element analysis of *HD-ZIP* genes in cabbage

Based on their physical locations in the gff3 file, 381 *HD-ZIP* family genes were mapped to chromosomes of four Chinese cabbage species, namely, *Brassica rapa* ssp. *pekinensis* A03 and *Brassica rapa* ssp. *pekinensis* Chiifu (heading ones) and *Brassica campestris* ssp. *chinensis* NHCC001 and *Brassica rapa* ssp. *chinensis* pakchoi (non-heading ones) (**Supplementary Figure S2**). Chromosome mapping showed that each cabbage cultivar contained 10 chromosomes, with chromosome A09 being the longest (50–70 Mb) and chromosomes A04/A10 the shortest (20–30 Mb). In addition to the 3 genes (*BraAng00243P*, *BraAng00244P*, and *BraAng00319P*) that were discovered on Ann, an A-genome chromosome that was randomly selected in *Brassica rapa* ssp. *chinensis* pakchoi, the remaining 378 genes were unevenly distributed on the chromosomes of the 4 cabbage cultivars; the quantity of *HD-ZIP* family genes on each chromosome varied from 6 to 14. Heading and non-heading cabbage did not show any *HD-ZIP III* genes on chromosomes A03 or A07; *Brassica rapa* ssp. *pekinensis* Chiifu did not show any *HD-ZIP III* genes on chromosome A09. There were more than 10 *HD-ZIP* family genes each on the A02, A07, and A09 chromosomes; the 4 cabbage cultivars had a completely same distribution of *HD-ZIP* genes on chromosome A04. Heading Chinese cabbage had a relatively narrower distribution of *HD-ZIP* genes on chromosomes A03 and A10 than non-heading cabbage. Specifically, heading Chinese cabbage had a smaller number of *HD-ZIP II* genes on chromosome A03 and a smaller number of HD-ZIP I genes on chromosome A10. There was an uneven distribution of these *HD-ZIP* genes in heading and non-heading cabbage as well as among the four species (**Supplementary Figure S2**).

**Figure 6:**
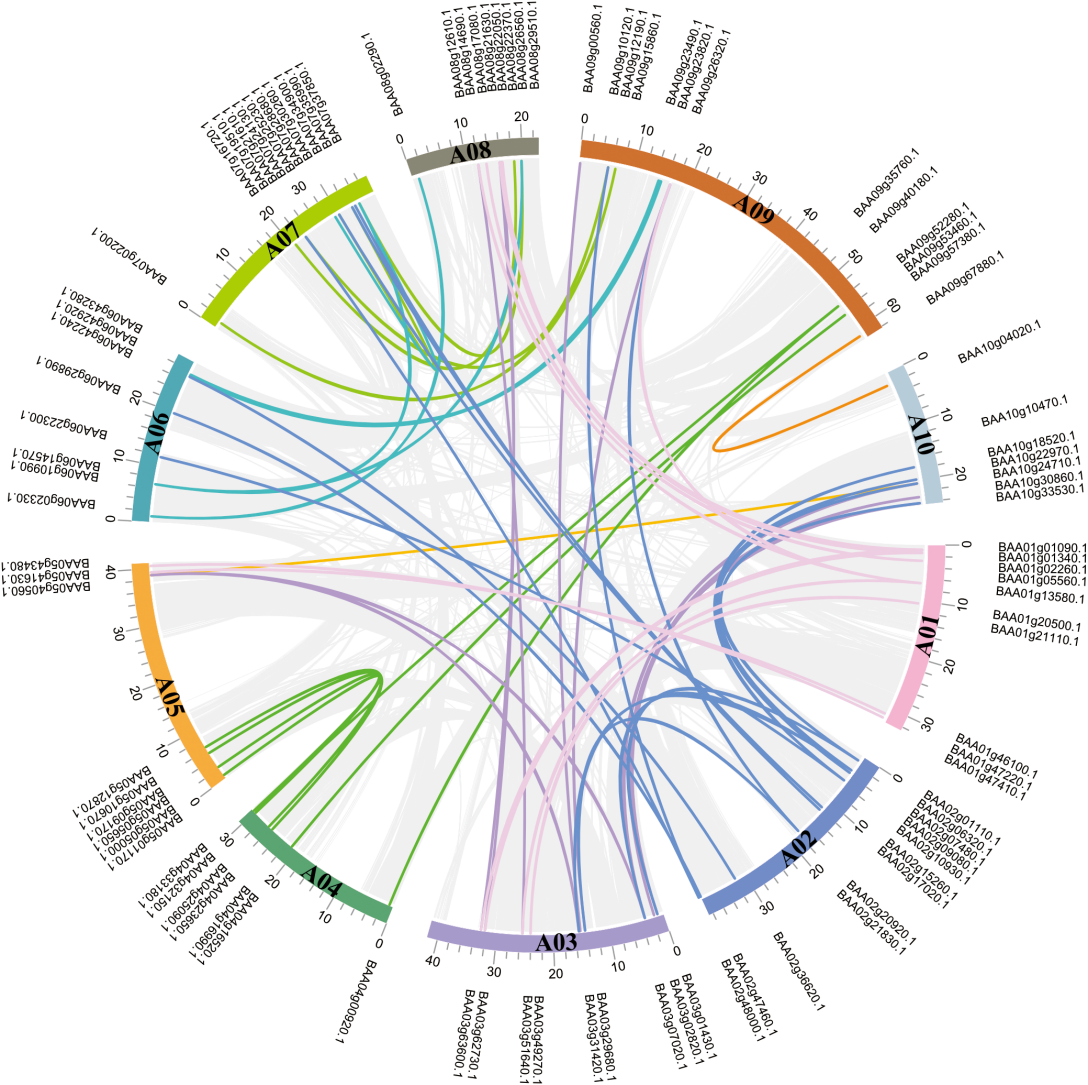
Identification of duplicated *HD-ZIP* gene pairs in *Brassica rapa* ssp. *pekinensis* A03. The lines of different colors showed the duplicated *HD-ZIP* gene pairs with collinearity relationships, the backdrop’s gray lines indicated the synteny blocks of the *HD-ZIPs* in the *Brassica rapa* ssp. *pekinensis* A03 genome.

Gene duplication is essential for generating novel functions and expanding gene families. Genes that are closely connected on the same chromosome and are less than 200 kb apart are tandem repeats; otherwise, they are segmental duplications (Bohutínská et al., 2021; Flagel and Wendel, 2009). We investigated *HD-ZIP* fragments and tandem repeat sequences in the genome of heading cabbage A03 using MCScan to further understand the amplification mechanism of *HD-ZIP* family genes. No tandem duplication events were discovered in the 95 *HD-ZIP* genes, but 61 pairs of fragment duplication events were detected and distributed across relevant chromosomes (**Figure 6**). Among these 61 co-linked pairings, 10 genes, such as *BAA02g10930*.*1*–*BAA06g22300*.*1* and *BAA02g10930*.*1*–*BAA10g18520*.*1*, *BAA02g01110*.*1*–*BAA10g33530*.*1*, and *BAA02g01110*.*1–BAA03g01430*.*1*, showed one-to-many pairing, indicating that many *HD-ZIP* genes in the Chinese cabbage A03 genome are homologous.

*Cis*-acting elements in the promoter regions of the *HD-ZIP* genes in *Brassica rapa* ssp. *pekinensis* A03 were identified using PlantCARE. In **Supplementary Figure S3**, the positions and numbers of all *cis*-acting elements are shown with different colored rectangles. In our analysis, *cis*-acting components were mainly divided into 17 categories: abscisic acid responsiveness, auxin responsiveness, gibberellin responsiveness, methyl jasmonate (MeJA) responsiveness, salicylic acid responsiveness, defense and stress responsiveness, drought inducibility, low-temperature responsiveness, wound responsiveness, differentiation of the palisade mesophyll cells, endosperm expression, meristem expression, seed-specific regulation, cell cycle regulation, circadian control, flavonoid biosynthetic gene regulation, and zein metabolism regulation (**Supplementary Figure S3**). Based on statistical analysis, the *cis*-acting element ‘MeJA responsiveness’ had the highest percentage among the promoter regions of the 4 HD-ZIP subfamilies, ranging from 22 to 30%, followed by ‘abscisic acid responsiveness’, with a range of 12–25% (**Supplementary Figure S4A**). Furthermore, in the promoter regions of the 4 *HD-ZIP* subfamilies, the percentage of the 5 *cis*-acting elements associated with plant hormones—abscisic acid, auxin, gibberellin, MeJA, and salicylic acid responsiveness—was between 64 and 68% (**Supplementary Figure S4B**).

#### 3.3.2 Interaction network of partial HD-ZIP proteins in *Brassica rapa* ssp. *pekinensis* A03

**Figure 7:**
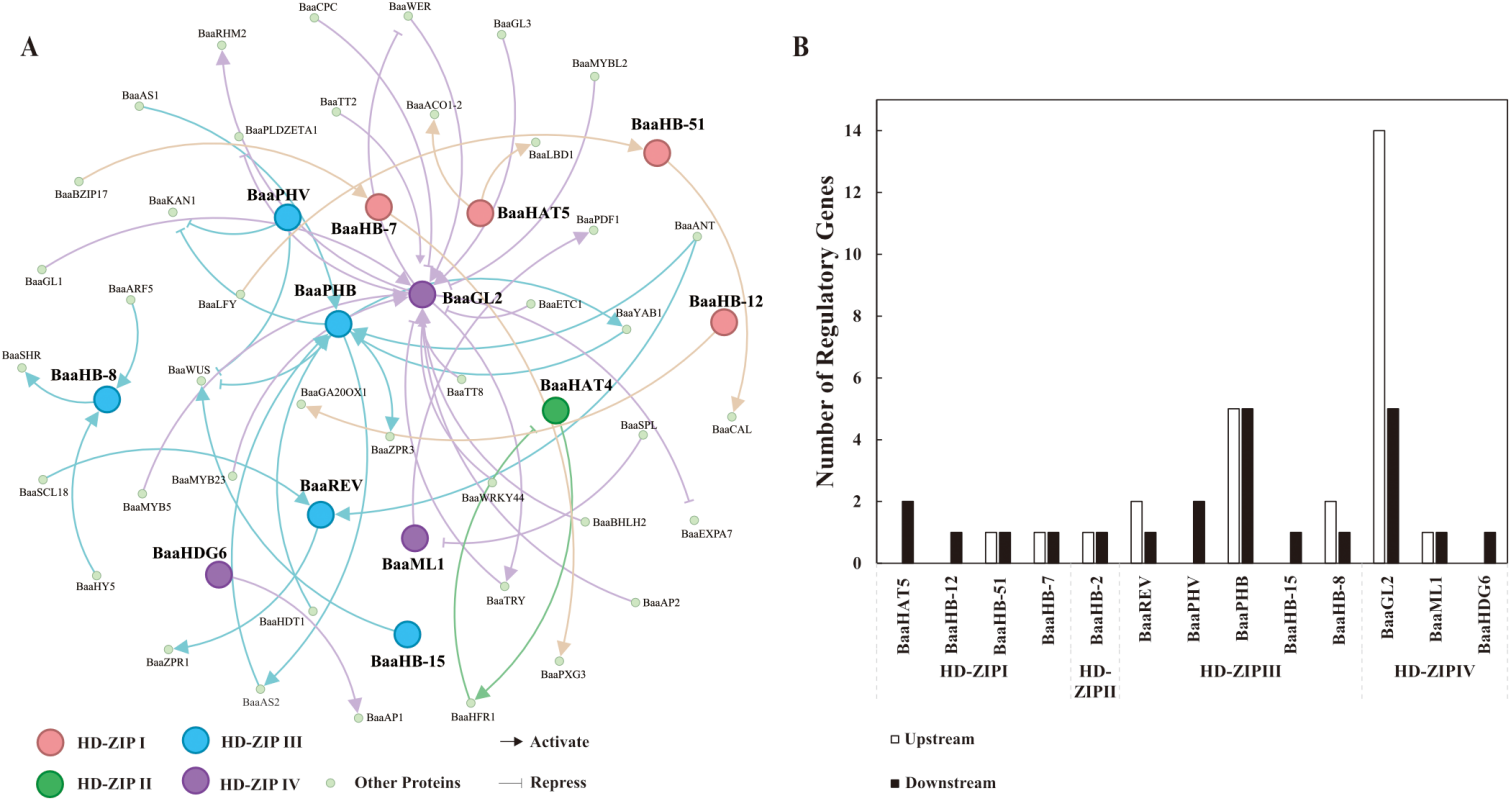
Prediction of upstream and downstream proteins and their interaction networks for the HD-ZIP proteins in *Brassica rapa* ssp. *pekinensis* A03. **(A)** Regulatory network between HD-ZIP proteins and their upstream and downstream regulatory proteins; **(B)** Number of upstream and downstream regulatory proteins for HD-ZIP proteins.

The protein regulatory network is an important strategy for investigating the functions of HD-ZIP family proteins. To construct this network, the ATRM database was first utilized to predict the upstream and downstream regulatory proteins of the HD-ZIP family in Chinese cabbage. Subsequently, an interaction network was created between each HD-ZIP protein and its upstream and downstream proteins to highlight their regulatory pathways. The ATRM database revealed 13 HD-ZIP family members that shared regulatory interactions with 41 additional proteins. The BaaGL2 protein of the HD-ZIP IV subfamily was connected with the largest number of regulatory proteins, with 14 upregulated and 5 downregulated. The HD-ZIP IV subfamily included two additional proteins: BaaML1 and BaaHDG6. BaaML1 exhibited one upstream and one downstream gene, whereas BaaHDG6 had only one downstream protein (**Figure 7**). In the HD-ZIP I subfamily, only BaaHB-1 and BaaHB-12 were predicted to have downregulated proteins, whereas BaaHB-51 and BaaHB-7 were predicted to have one upstream and one downstream proteins, respectively. For BaaHB-2 from the HD-ZIP II subfamily, one upstream and one downstream protein were predicted (**Figure 7**). All five members of the HD-ZIP III subfamily had putative regulatory proteins. BaaPHB was predicted to have five upstream and one downstream protein, whereas BaaREV and BaaHB-8 predicted two upstream and one downstream protein. For BaaPHV and BaaHB-15, only proteins downstream of them were predicted, with two for BaaPHV and one for BaaHB-15 (**Figure 7**). As illustrated in **Figure 7**, BaaAS1, BaaAS2, BaaYAB1, BaaANT, and BaaHDT1 may positively regulate BaaPHB, which in turn may enhance the expression of BaaAS2, BaaYAB1, and BaaZPR3 (**Figure 7B**).

#### 3.3.3 Regulatory network prediction and GO analysis for *HD-ZIP*-related genes with distinct expression

**Figure 8:**
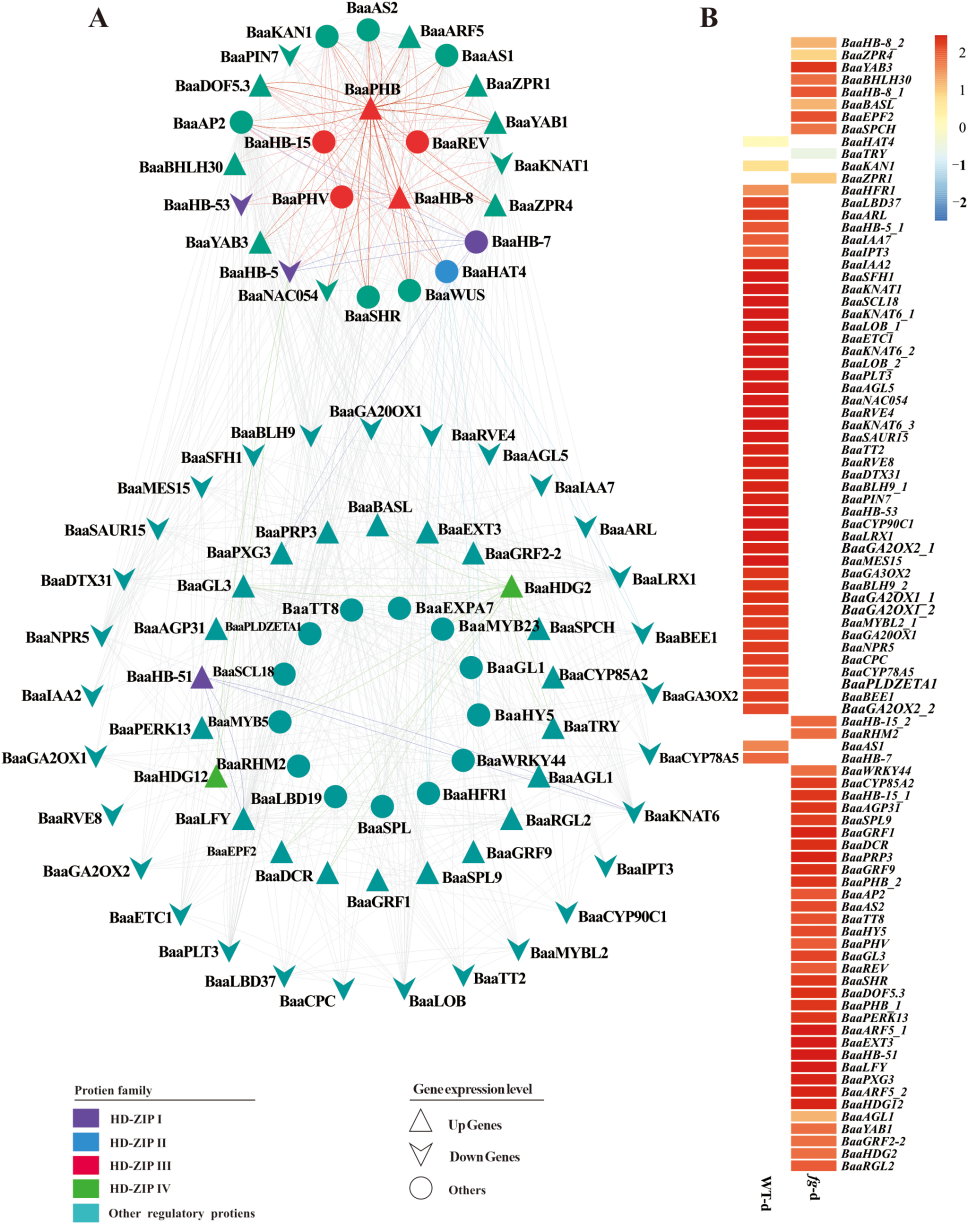
Regulation network prediction and expression heatmap of genes associated with *HD-ZIP.* **(A)** A functional protein association network was predicted by STRING (https://cn.string-db.org/); **(B)** Gene differential expression in *fg-d* and *WT-d* was represented using heatmaps (log_2_FC ≥ 1 or ≤ −1).

Heading is a key agronomic trait in Chinese cabbage. To analyze the molecular biology functions of the HD-ZIP family in heading Chinese cabbage, we comparatively analyzed RNA sequencing (RNA-seq) data between a non-heading mutant (*fg-1*) and the heading wild-type A03 (WT) (Li et al., 2019b). The non-heading mutant *fg-1* was isolated from the EMS-induced mutant population derived from the heading Chinese cabbage inbred line A03 (Li et al., 2019b). The differentially expressed proteins that clustered with the HD-ZIP family (orange box in **Supplementary Figure S5**) and the predicted upstream and downstream regulatory network proteins of the HD-ZIP family (**Figure 7**) were evaluated using STRING (Functional Protein Association Networks) analysis based on the results of RNA-seq. A functional protein association network of 131 proteins (**Supplementary Table S5**) was constructed and visualized in **Figure 8A**. Twelve proteins belonging to the HD-ZIP family were found in this network. These proteins included four from the HD-ZIP I subfamily (BaaHB-7, BaaHB-53, BaaHB-5, and BaaHB-51), one from the HD-ZIP II subfamily (BaaHAT4), five from the HD-ZIP III subfamily (BaaREV, BaaPHB, BaaPHV, BaaHB-15, and BaaHB-8), and two from the HD-ZIP IV subfamily (BaaHDG2 and BaaHDG12). There were 25 proteins directly associated with HD-ZIP III. Fourteen proteins had functional interactions with BaaPHB; of these, BaaYAB1, BaaYAB3, BaaARF5, BaaZPR1, BaaZPR4, and BaaDOF5.3 were up-regulated in *fg*-d and BaaKNAT1 and BaaNAC054 were down-regulated (log_2_FC ≥ 1 or ≤ −1) (**Figure 8B**).

#### 3.3.4 Enrichment analysis of genes in the regulation network

**Figure 9:**
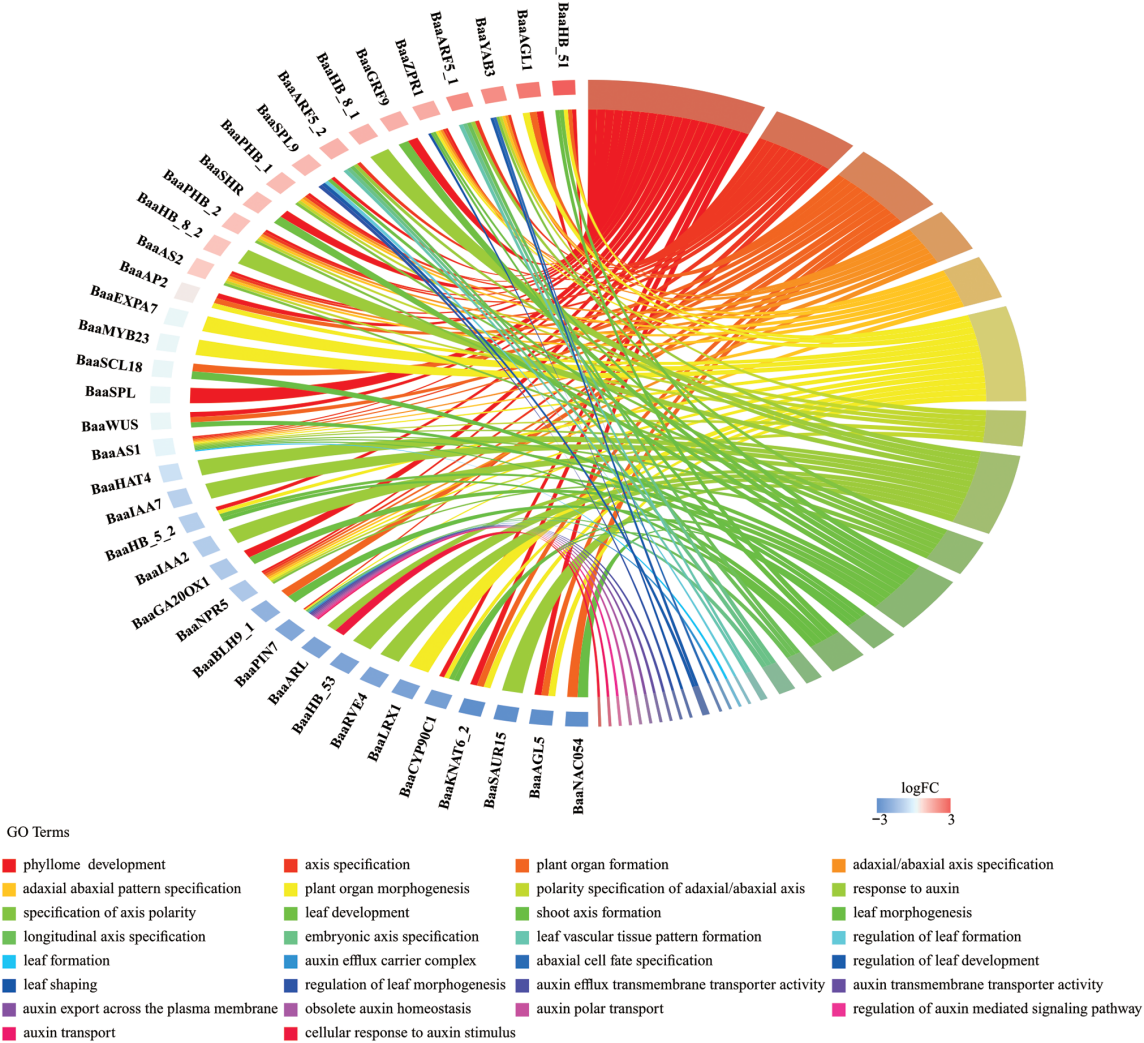
GO chord plot. In the chord diagram, genes were represented on one side of the circle, and GO terms were represented on the opposite side. The arcs (ribbons) connecting genes and GO terms indicated the strength of the association, with thicker ribbons representing stronger relationships.

The 131 genes in the regulation network (**Figure 8**) were subjected to GO analysis to comprehend the overall biological processes or associated functional traits. As shown in **Figure 9**, a total of 38 genes were enriched in 30 pathways. These pathways included 9 associated with leaf development, 10 with auxin, 9 with polarity, and 2 with plant organ development. Eleven pathways contained five or more genes each, including phyllome development, plant organ morphogenesis, response to auxin, plant organ formation, axis specification, leaf development, adaxial/abaxial axis specification, adaxial/abaxial pattern specification, polarity specification of adaxial/abaxial axis, specification of axis polarity, and leaf morphogenesis (**Figure 9**). As shown in **Figure 9**, there were nine biological processes involved in leaf development: phyllome development, regulation of leaf morphogenesis, leaf vascular tissue pattern formation, regulation of leaf formation, leaf development, leaf shaping, regulation of leaf development, leaf formation, and leaf morphogenesis.

## 4 Discussion

### 4.1 Origins and conservation of plant HD-ZIP transcription factors

The HD-ZIP transcription factors, which play a pivotal role in plant development, have evolved gradually and are closely associated with the diversification of plant species. To elucidate the evolutionary origins of HD-ZIPs, we analyzed genomic data from 11 algal species, categorized into Rhodophyta, Chlorophyta, and Charophyta. While Rhodophyta and Chlorophyta exhibit relatively simple morphological structures lacking distinguishable organs such as roots, stems, and leaves, Charophyta displays organ differentiation resembling these structures. Our findings revealed the absence of HD-ZIP proteins in *Galdieria sulphuraria* and *Chondrus crispus* of Rhodophyta as well as in *Micromonas pusilla*, *Ostreococcus lucimarinus*, *Coccomyxa subellipsoidea*, *Chlorella variabilis*, *Monoraphidium neglectum*, *Chromochloris zofingiensis*, *Volvox carteri*, and *Chlamydomonas reinhardtii* of Chlorophyta (**Figure 1**). These results align with those of Mukherjee et al. (2009), who reported the absence of HD-ZIP proteins in three green algae (*Chlamydomonas reinhardtii*, *Ostreococcus lucimarinus*, and *Ostreococcus tauri*) and one red alga (*Cyanidioschyzon merolae*). In contrast, we identified three HD-ZIP proteins—one each from HD-ZIP I, HD-ZIP II, and HD-ZIP III—in *Charophyta braunii* (Charophyta). This finding aligned with previous studies, such as that of Zalewski et al. (2013), who discovered HD-ZIP IV subfamily proteins in *Coleochaete orbicularis* and *Spirogyra pratensis*, and Wang et al. (2020), who identified HD-ZIP transcription factors in *Mesostigma viride* and *Chlorokybus atmophyticus*. Collectively, these results supported the hypothesis that HD-ZIP proteins originated in Charophyta, coinciding with the emergence of root-, stem-, and leaf-like differentiation.

In higher plant groups, including bryophytes, pteridophytes, gymnosperms, and angiosperms, HD-ZIP family genes were identified in the genomes of 76 species. These encompassed bryophytes (*Physcomitrella patens* and *Marchantia polymorpha*), pteridophytes (*Selaginella moellendorffii*), gymnosperms (*Ginkgo biloba*), basal angiosperms (*Nymphaea colorata* and *Amborella trichopoda*), and angiosperms (eudicots and monocots). Each species contained genes from all four HD-ZIP subfamilies: HD-ZIP I, HD-ZIP II, HD-ZIP III, and HD-ZIP IV (**Figure 1**). These findings are consistent with previous studies, such as Sakakibara et al. (2001), who identified 11 HD-ZIP genes in the fern *Ceratopteris richardii* and 10 in the moss *Physcomitrella patens*, and Mukherjee et al. (2009), who reported HD-ZIP genes in six plant species ranging from mosses to angiosperms. Yip et al. (2016) further identified HD-ZIP III subfamily genes in four mosses (*Physcomitrella patens*, *Dawsonia superba*, *Ceratodon purpureus*, and *Sphagnum* sp.). Our study expanded on these findings by identifying the number of genes in the four HD-ZIP subfamilies across 87 species and presenting them alongside a phylogenetic tree. This comprehensive analysis not only broadens our understanding of HD-ZIP gene distribution but also highlights the relationship between species’ evolutionary distance, clades, and the number of HD-ZIP genes.

### 4.2 Regulatory roles of *HD-ZIP* genes in leaf development and Chinese cabbage head formation

The leafy head of Chinese cabbage, a key agronomic trait, significantly influences its quality and economic value. However, heading is a quantitative trait controlled by multiple genes and susceptible to environmental factors, making it challenging to study (Zhang et al., 2022). *HD-ZIP* genes are known to regulate leaf growth, development, and morphogenesis. For instance, in the *HD-ZIP I* subfamily, *ATHB13* modulates *Arabidopsis* leaf width (Hanson et al., 2001), while overexpression of *HaHB-4* results in rounder leaves (Dezar et al., 2005). In the *HD-ZIP II* subfamily, *35S::HAT2* transgenic plants exhibit smaller leaves (Sawa et al., 2002). Within the *HD-ZIP III* subfamily, *PHABULOSA* (*PHB*) and *PHAVOLUTA* (*PHV*) regulate leaf adaxial/abaxial polarity during *Arabidopsis* leaf primordium development, with dominant mutations in *phb* and *phv* causing a shift from abaxial to adaxial fates (McConnell et al., 2001). In Chinese cabbage, *BcpLH* regulates leaf polarity through the miR165/166–*HD-ZIP III* module (Ren et al., 2020), and HD-ZIP III transcription factors are highly expressed on the adaxial side (Sun et al., 2024). Comparative analyses of heading traits in Chinese cabbage have identified candidate genes related to domestication, including those involved in leaf polarity determination, such as *ARFs*, *KANADIs*, and *HD-ZIP III* (Cheng et al., 2016; Bohutínská et al., 2021). However, the specific regulatory mechanisms of HD-ZIP III subfamily proteins in Chinese cabbage leafy head development remain poorly understood. Elucidating these mechanisms is critical for advancing molecular breeding strategies to enhance yield.

To address this, our study conducted the following investigations:

1. Evolutionary and Developmental Analysis: We examined the evolutionary and developmental relationships of HD-ZIP proteins, focusing on the highly conserved domains and motifs of the HD-ZIP III subfamily, using 22 Brassicaceae species as representatives.
2. Pan-Genome Analysis: In the pan-genome A of 18 *Brassica rapa* species, we observed that although the protein sequences and domains of HD-ZIP III were highly conserved, differences were identified in the auxin-related cis-elements of the HD-ZIP III promoter regions between heading and non-heading cabbage. This finding suggested that the molecular function of HD-ZIP III was differentially regulated by auxin in heading and non-heading cabbages.
3. Regulatory Network Analysis: Transcriptional data, regulatory network prediction, GO enrichment, and qRT-PCR were employed to identify key genes interacting with HD-ZIP III in cabbage heading formation. Using a non-heading cabbage mutant (*fg-1*) derived from heading cabbage A03 (WT) by EMS mutagenesis, RNA-seq analysis revealed differentially expressed genes, primarily concentrated between WT-d and *fg-d* samples. Among these, 131 genes formed a protein interaction network (**Figure 8**) and clustered with HD-ZIP family genes (**Supplementary Figure S5**). Heatmap analysis indicated concentrated gene aggregation within the HD-ZIP III subfamily. GO enrichment revealed 38 genes (including HD-ZIP genes and differentially expressed genes) concentrated in three major pathways: auxin response, leaf development, and polarity regulation.

A comprehensive network analysis identified five HD-ZIP III subfamily members (*BaaPHV*, *BaaHB-8*, *BaaPHB*, *BaaHB-15*, and *BaaREV*) forming a regulatory module with six genes: *BaaAS1*, *BaaAS2*, *BaaARF5*, *BaaWUS*, *BaaZPR1*, and *BaaSCL18*. GO enrichment indicated that *BaaAS1* and *BaaARF5* were enriched in auxin response, leaf development, and polarity regulation, while *BaaAS2*, *BaaWUS*, and *BaaZPR1* were enriched in leaf development and polarity regulation. *BaaSCL18* was specifically associated with polarity-related pathways.

Three regulatory pathways were predicted and proposed (**Supplementary Figure S6**): (1) *BaaPHB* activated *BaaAS1* expression, while *BaaARF5* activated *BaaHB-8* expression, jointly participating in auxin accumulation, leaf development, and polarity regulation; (2) *BaaPHV*, *BaaPHB*, and *BaaHB-15* inhibited *BaaWUS* expression, while *BaaPHB* activated *BaaAS2*, forming a feedback loop. Additionally, *BaaREV* activated *BaaZPR1*, contributing to leaf development and polarity regulation; (3) *BaaSCL18* activated *BaaREV*, participating in polarity regulation.

**Figure 10:**
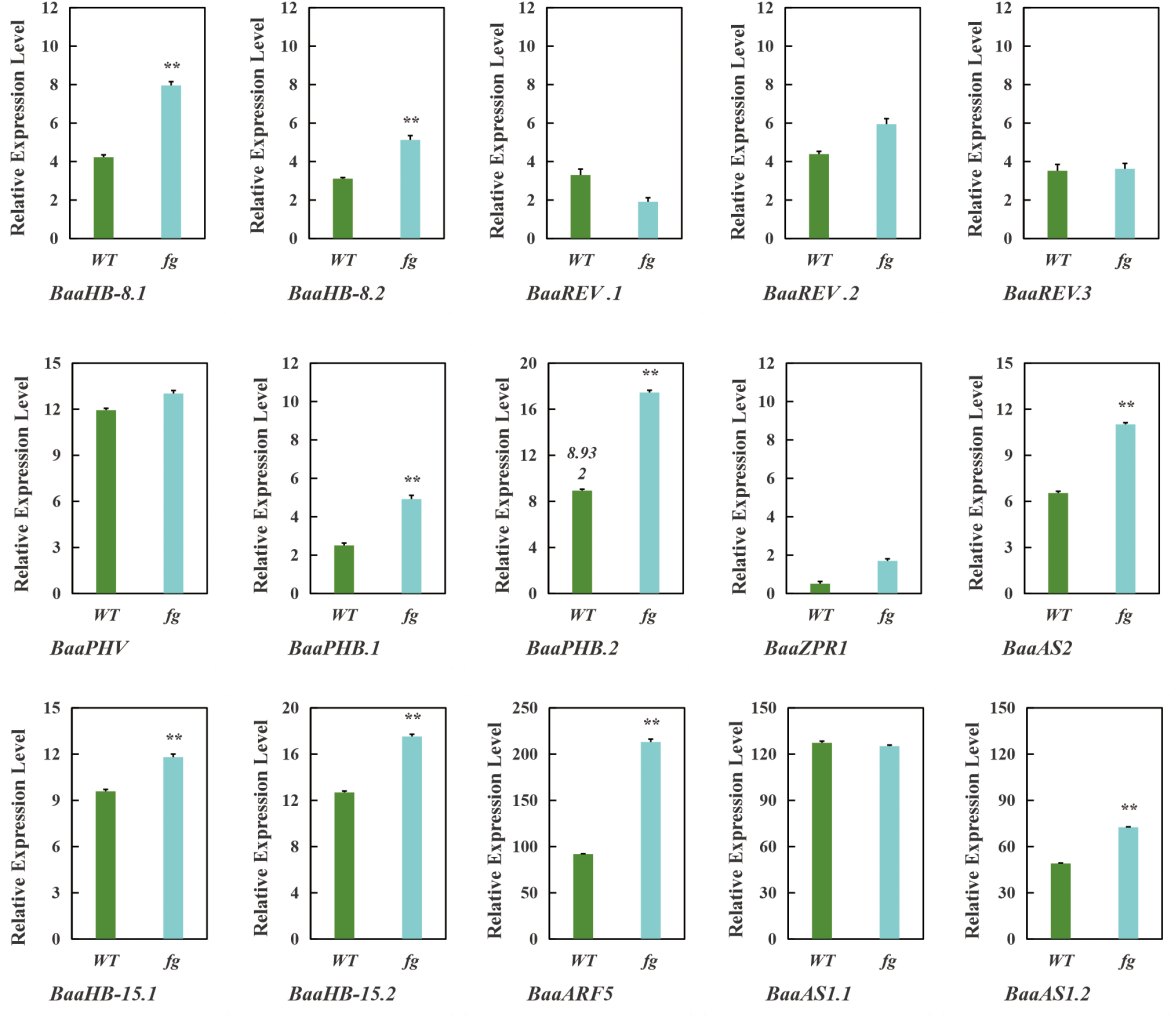
qRT-PCR analysis of genes in mutant (*fg-1*) and wild-type A03 (WT). Data were shown as the averages of three independent biological experiments (three technical replicates were taken for each biological experiment), and error bars indicated the SDs from three independent experiments. **P<0.01.

qRT-PCR results confirmed that nine genes (*BaaHB-8.1*, *BaaHB-8.2*, *BaaPHB.1*, *BaaPHB.2*, *BaaAS2*, *BaaAS1.2*, *BaaHB-15.1*, *BaaHB-15.2*, and *BaaARF5*) were significantly upregulated in the mutant *fg-1* compared to wild-type A03, while six genes (*BaaREV.1*, *BaaREV.2*, *BaaREV.3*, *BaaPHV*, *BaaZPR1*, and *BaaAS1.1*) showed no significant changes in expression (**Figure 10**). These results align with the proposed regulatory model, providing mechanistic insights into HD-ZIP III-mediated regulation of leafy head formation in Chinese cabbage.

In conclusion, our study elucidates the evolutionary origins of HD-ZIPs, their conservation across plant lineages, and their regulatory roles in Chinese cabbage leafy head development. These findings establish a fundamental basis for subsequent molecular breeding endeavors aimed at enhancing crop yield and quality.

## Data Availability

The original contributions presented in the study are included in the article/**Supplementary Material**. Further inquiries can be directed to the corresponding authors.

## Supplementary material

Supplementary Figure 1 Prediction of evolutionary timescales for all plant species involved in this study.

Supplementary Figure 2 Chromosome locations of *HD-ZIP* genes in heading and non-heading cabbage.

Supplementary Figure 3
*Cis*-acting regulatory elements **(A)** and their distribution in *HD-ZIP* gene promoters **(B)** in *Brassica rapa* ssp. *pekinensis* A03.

Supplementary Figure 4 Proportion of *cis*-acting elements in the promoter regions of four HD-ZIP subfamilies.

Supplementary Figure 5 Differential gene heatmap between the mutant *fg-1* and A03 (WT) (log2 FC≥1 or ≤−1).

Supplementary Figure 6 Model of HD-ZIP III participating in the regulation of leafy head formation in Chinese cabbage.
