# Supplementary figures and images for: Evolution of HD-ZIP transcription factors and their function in cabbage leafy head formation
Source: Front Plant Sci. 2025 Apr 3;16:1583110. doi: 10.3389/fpls.2025.1583110 (PMC12003273; doi:10.3389/fpls.2025.1583110)

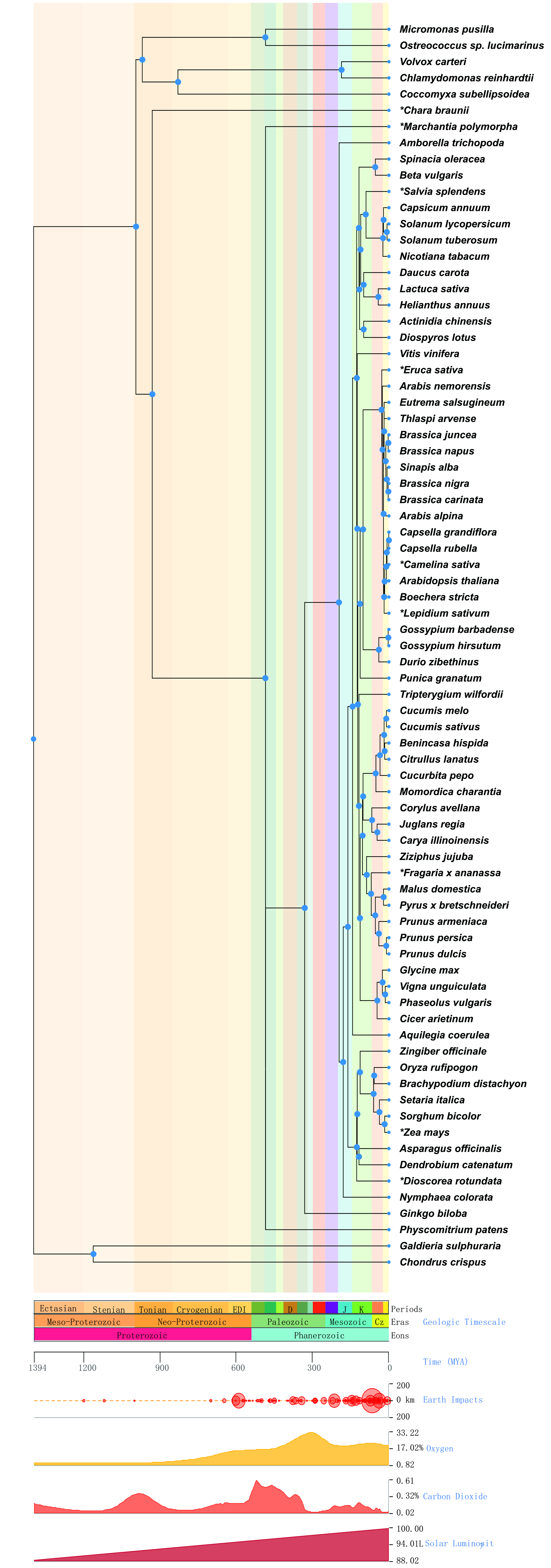

Supplement: Supplementary Figure 1 — Prediction of evolutionary timescales for all plant species involved in this study. [file DataSheet1.zip › Corrective supplementary materials/Supplementary Figure S1. Prediction of evolutionary timescales for all plant species involved in this study.tif]

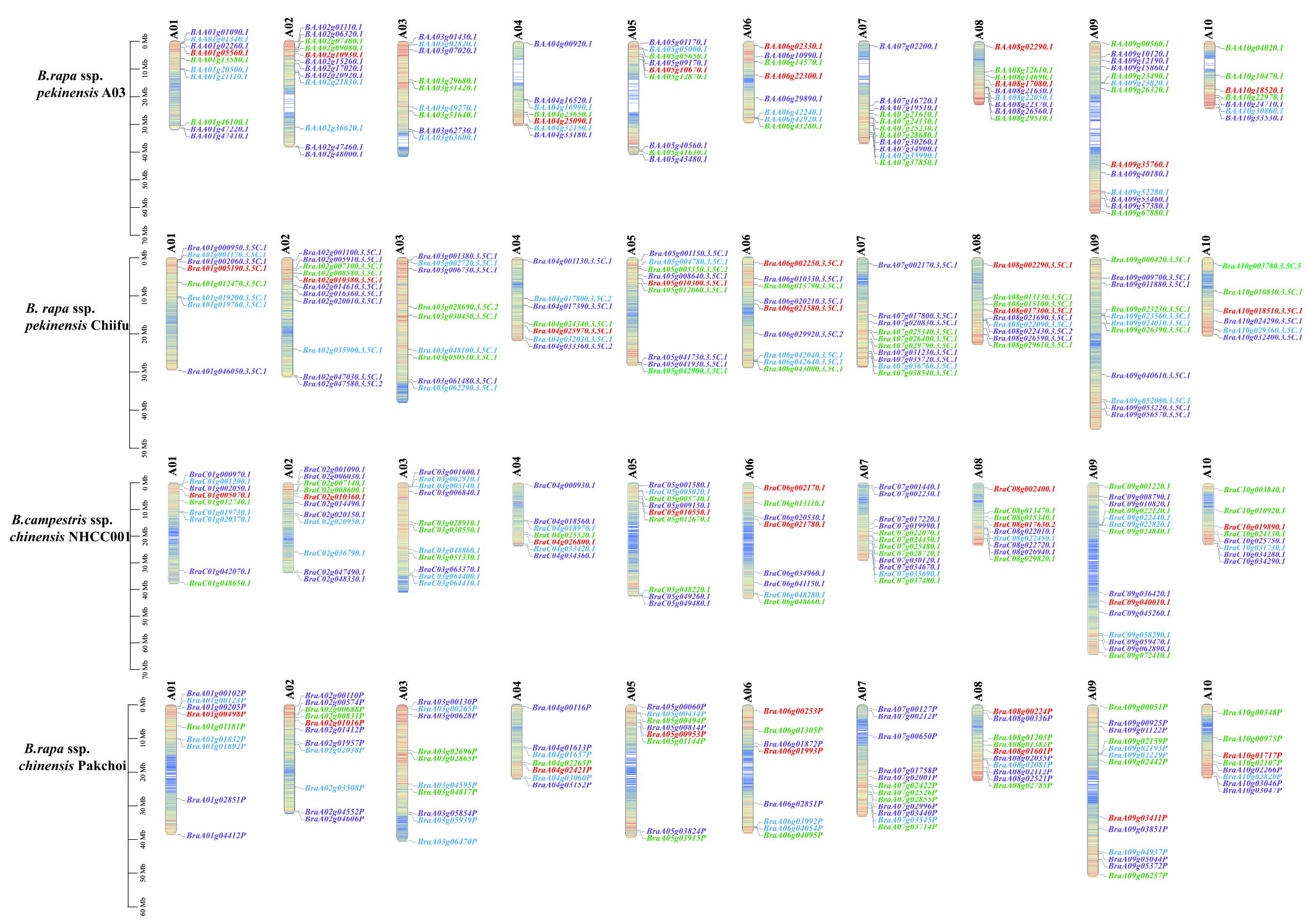

Supplement: Supplementary Figure 1 — Prediction of evolutionary timescales for all plant species involved in this study. [file DataSheet1.zip › Corrective supplementary materials/Supplementary Figure S2. Chromosome locations of HD-ZIP genes in heading and non-heading cabbage.tif]

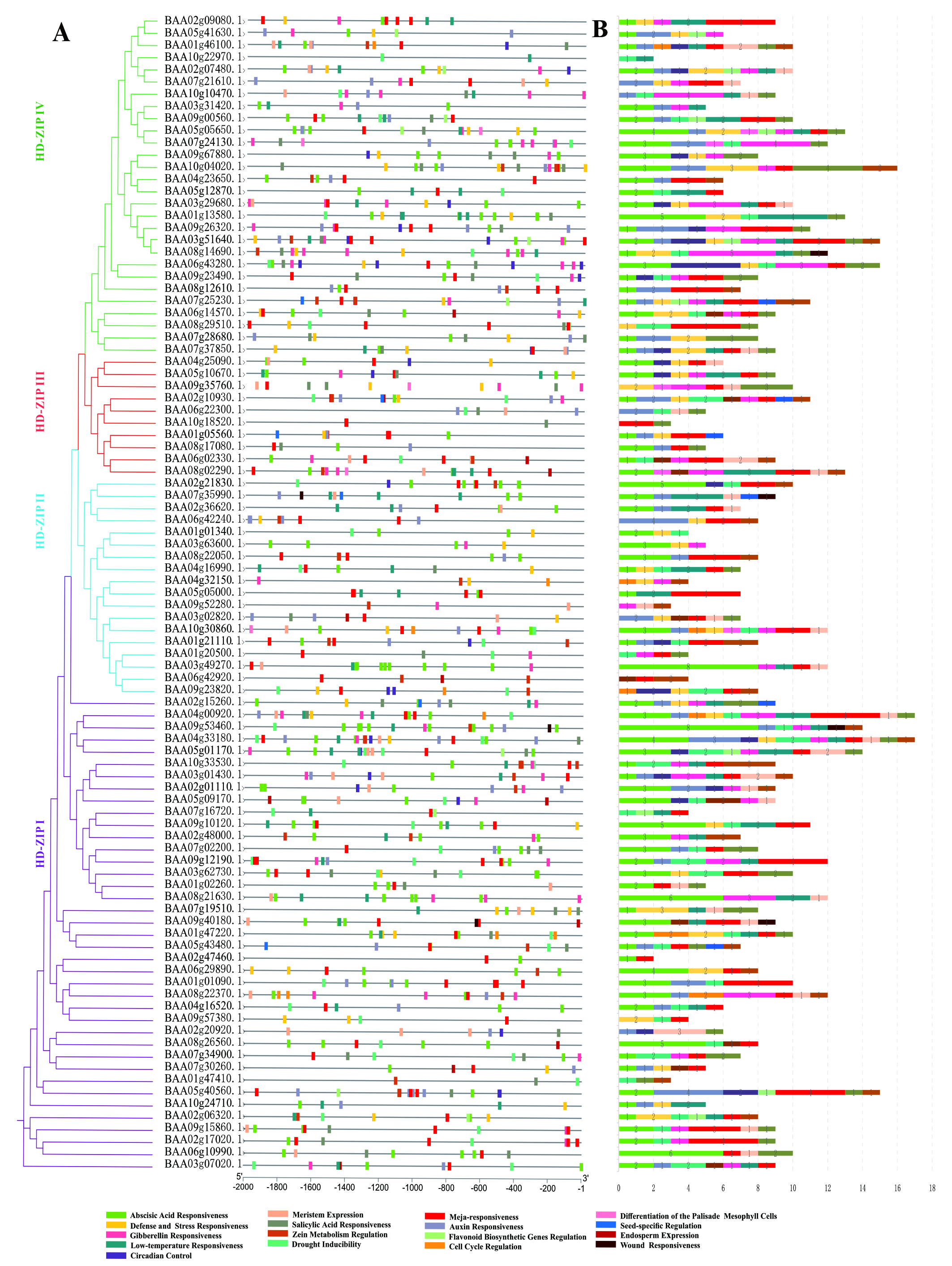

Supplement: Supplementary Figure 1 — Prediction of evolutionary timescales for all plant species involved in this study. [file DataSheet1.zip › Corrective supplementary materials/Supplementary Figure S3 Cis-acting regulatory elements (A) and their distribution in HD ZIP gene promoters (B) in Brassica rapa ssp pekinensis A03.tif]

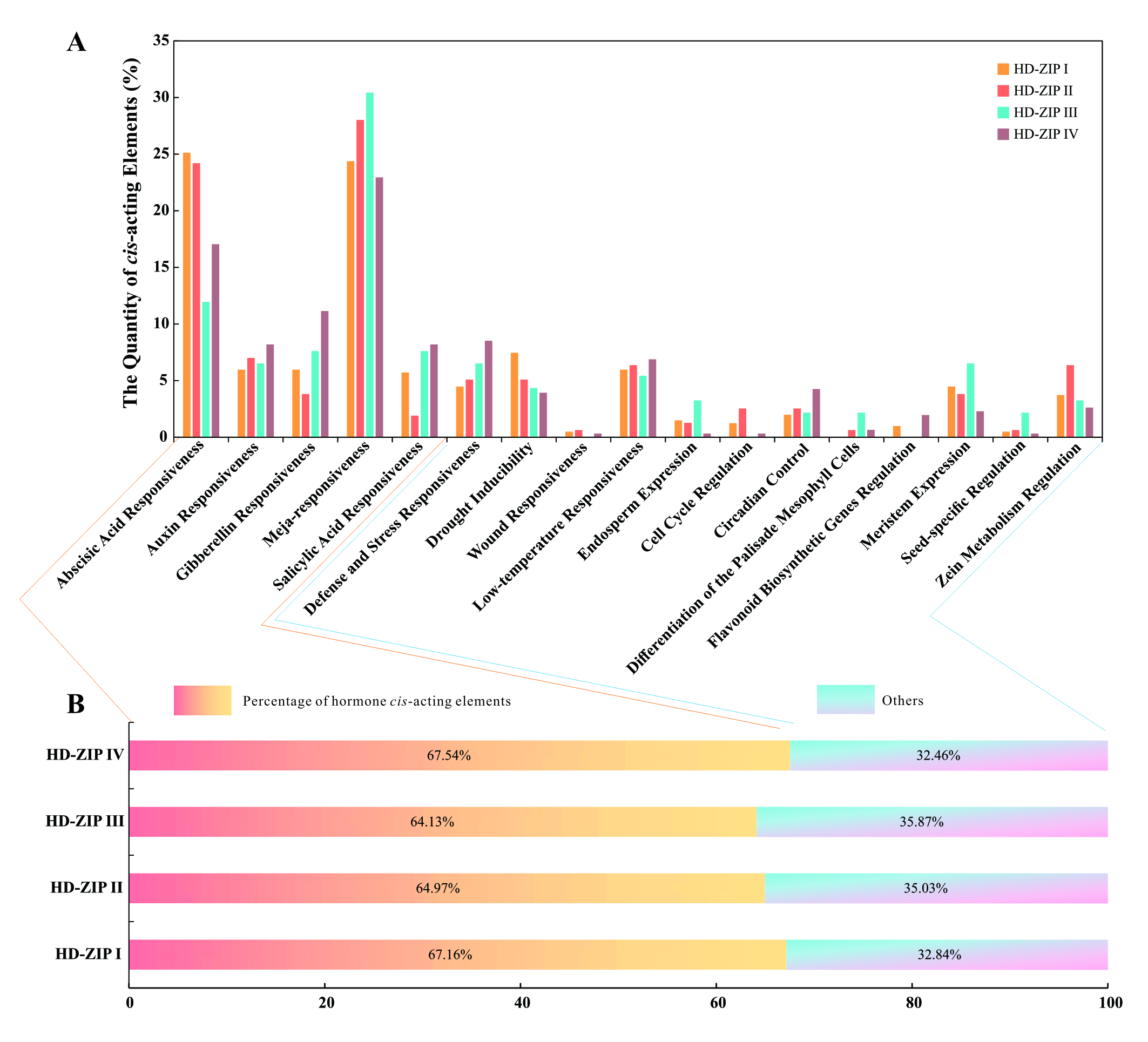

Supplement: Supplementary Figure 1 — Prediction of evolutionary timescales for all plant species involved in this study. [file DataSheet1.zip › Corrective supplementary materials/Supplementary Figure S4 Proportion of cis-acting elements in the promoter regions of four HD-ZIP subfamilies.tif]

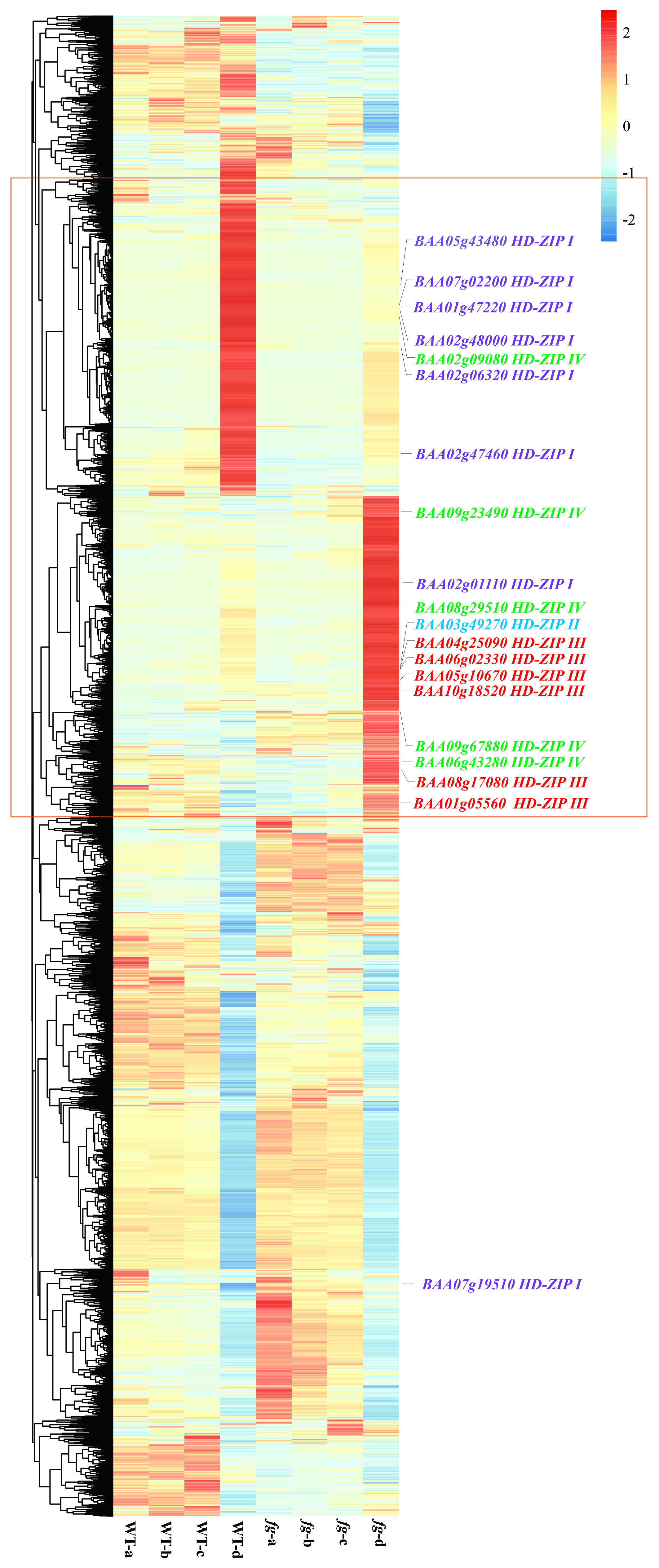

Supplement: Supplementary Figure 1 — Prediction of evolutionary timescales for all plant species involved in this study. [file DataSheet1.zip › Corrective supplementary materials/Supplementary Figure S5. Differential gene heatmap between the mutant fg-1 and the wild-type A03 (WT).tif]

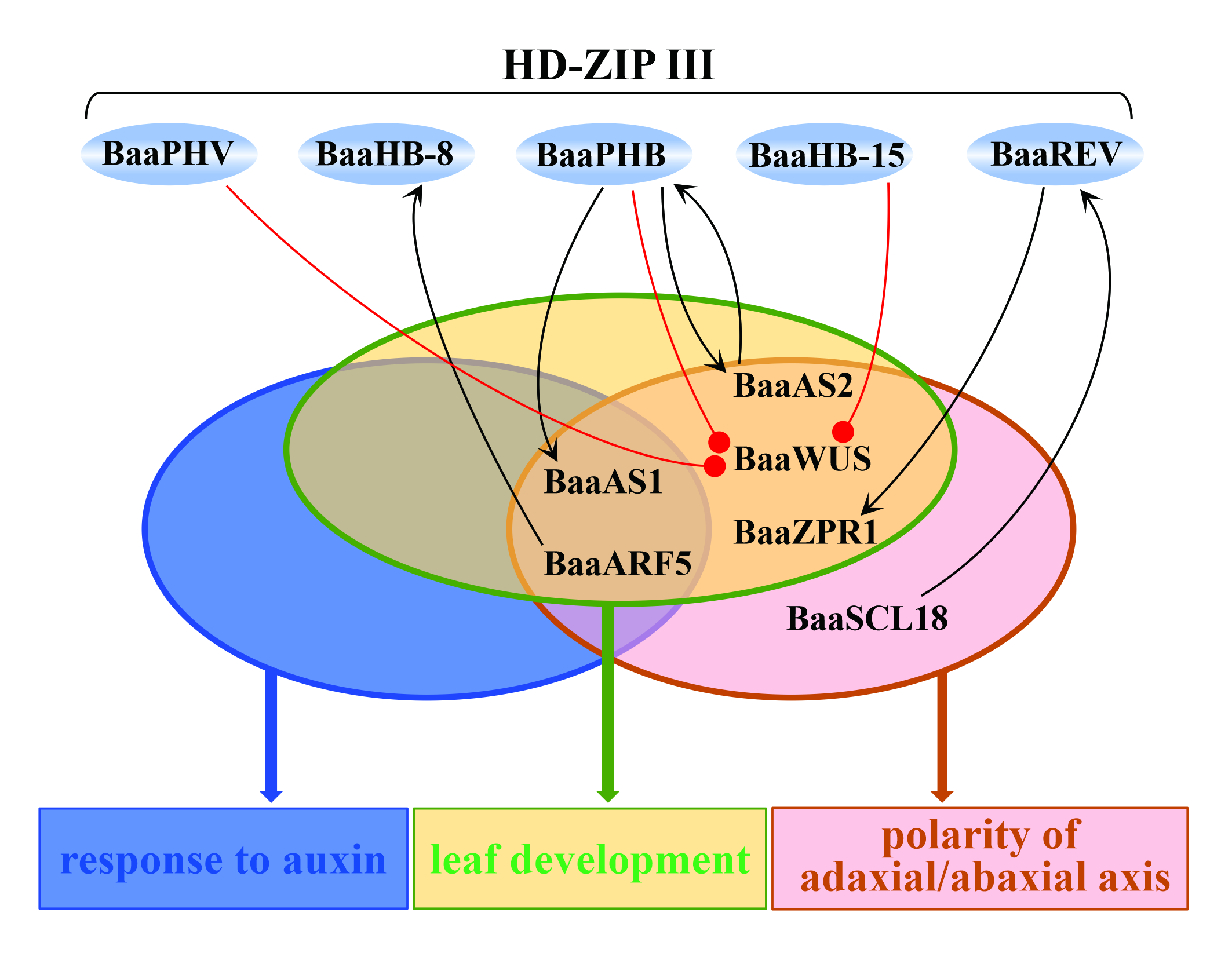

Supplement: Supplementary Figure 1 — Prediction of evolutionary timescales for all plant species involved in this study. [file DataSheet1.zip › Corrective supplementary materials/Supplementary Figure S6. Model of HD-ZIPIII participating in the regulation of leafy head formation in Chinese cabbage.tif]
